# Integrated risk evaluation of in- and outputs, including heavy metals, in broiler farm environments for the appearance of antimicrobial resistance

**DOI:** 10.1016/j.psj.2026.106819

**Published:** 2026-03-18

**Authors:** Theresa M Liegsalz, Mykhailo Savin, Uwe Blum, Céline Heinemann, Julia Steinhoff-Wagner

**Affiliations:** a Professorship of Animal Nutrition and Metabolism, Technical University of Munich, Freising, Germany; b Institute of Hygiene and Public Health, University Hospital Bonn, Bonn, Germany; c Bavarian State Institute for Forests and Forestry, Freising, Germany; d Institute of Animal Sciences, University of Bonn, Bonn, Germany; e HEF World Agricultural System Center, Technical University of Munich, Freising, Germany

**Keywords:** Chicken, Nitrogen fraction, Trace elements, Microorganism, Farm assessment

## Abstract

Although recommendations for nutritional factors have already been studied, derived and published, nutrients like trace elements continue to be much higher supplemented in broiler feed than required. If these nutrients, such as heavy metals as trace elements, are excreted due to an oversupply, they might accumulate in the environment and cause a major impact due to the risk of antibiotic-resistant co-selection. The aim of this study was to evaluate the accumulation of these nutritional factors in environmental samples, and their influential risk for the occurrence of antibiotic-resistant opportunistic pathogens. Samples from fresh feed and bedding, water from the water line, and manure and dust from broiler houses were collected during the last week of the fattening period from 14 German broiler farms. The pH of water, feed, bedding and manure, the water turbidity and electric conductivity, and nutritional (crude ash (**CA**), crude protein (**CP**), metabolic nitrogen (**N_met_**), crude fat), and fiber components (neutral detergent fiber, acid detergent fiber, acid detergent lignin) from feed, bedding and manure, as well as elements (*n* = 31) from all 5 sample types were analyzed. Additionally, the water, manure and dust were tested for the occurrence of methicillin-resistant *Staphylococcus aureus* and species with resistance to extended-spectrum beta-lactams. Group differences of the chemical analyses between the sample types and the risk of antibiotic-resistant, opportunistic microorganisms was evaluated. An accumulation of moisture, CA and CP could be observed from the feed and bedding to the manure samples, and in elements, from feed, bedding and water to manure and dust. The risk evaluation indicated a lower risk of antibiotic-resistant, opportunistic pathogens with higher concentrations of fiber in bedding or manure, and a higher risk with higher relative amounts of N_met_ in manure. Therefore, especially high fiber in bedding and low CP in feed could be relevant parameters for improving the health status of broilers, and N_met_ in manure could serve as a parameter for evaluating the environmental impact.

## Introduction

The broiler meat production is an open system embedded in the holistic agricultural cycle. Thus, inputs in the broiler fattening systems, such as day-old chicks, feed, fresh bedding materials or water, are influential for the outputs, such as the grown broiler and the manure. Manure, as an output and used as fertilizer, is influential for the quality of the soil (Gupta and Charles, 1999; Parente et al., 2018), which could lead to the risk of accumulating aversive substances in the soil, a negative impact on crop production, and, in turn, food and feed. Sheppard and Sanipelli (2012) showed a manure-to-feed ratio between 1.6 and 5.5 in 32 analyzed elements of poultry production farms (broiler, layer, turkey). These elements further accumulate in the soil when using the manure as fertilizer, as indicated by Gupta and Charles (1999) or Parente et al. (2018).

One major problem associated with these accumulations is the risk of antibiotic-resistant, opportunistic microorganisms, and additionally, the co-selection with heavy metals, originated in the animals' barns or manure and spread in the environment, such as soil or water (Seiler and Berendonk, 2012; Shen et al., 2023). In this context, the differentiation between co- and cross-resistance has to be noted. Co-resistance is, therefore, a resulted resistance against various agents due to the location of these mechanisms on the genetic element, whereas cross-resistance occurs if the resistance mechanism acts nonspecifically against the agents (Seiler and Berendonk, 2012; Yu et al., 2017). Various heavy metals, which are influential for this co-selection, were stated by various studies, where the most relevant might be arsenic (**As**), cadmium (**Cd**), cobalt (**Co**), copper (**Cu**), chromium (**Cr**), mercury (**Hg**), manganese (**Mn**), nickel (**Ni**), lead (**Pb**) and zinc (**Zn**) (Seiler and Berendonk, 2012; Nguyen et al., 2019; Mustafa et al., 2021; Shen et al., 2023). Not only because of the risk of co-selection, but also because high concentrations of heavy metals could be toxic for animals, like broilers (Aljohani, 2023), and humans or pose a harm for the environment, upper limits have been defined in the Commission Regulation (EU) No 1275/2013 for As (2 ppm), Cd (0.5 ppm) and Pb (5 ppm) in feed. However, some heavy metals are also essential trace elements for broilers. That is why the National Research Council (1994) published recommendations for dietary concentrations of, e.g., Cu (8 ppm), Mn (80 ppm) and Zn (40 ppm) in feed (90% dry matter, **DM**) based on conducted dose-response studies, like for Zn in Steinruck and Kirchgessner (1993). Additionally, for food productions, the established Maximum Residue Limits must be adhered to Regulation (EC) No. (1831/2003), leading to a necessary limit of such trace elements in the livestocks’ feed.

Another considerable aspect should be two nutritional fractions, such as crude protein (**CP**) or fiber. Excesses of the contained N can be found as uric acid and ammonium in the excreta (Such et al., 2021) and emitted as ammonia into the environment (van Harn et al., 2012; de Rauglaudre et al., 2025). In this context, relationships between ammonia stress and resistance mechanisms have already been discussed for aerobic digested manure by Zhang et al. (2020) and for fertilized soil by Sun et al. (2020). Furthermore, Liegsalz et al. (2025) showed positive correlations between fiber components in bedding materials and the water holding capacity (**WHC**), which could reduce the microbiological load in the broilers’ environment as indicated in Gontar et al. (2022). Therefore, this study aimed to conduct a benchmark between farms, evaluating environmental samples from broiler houses by the accumulation of nutritional factors, fiber components and elements, like trace elements or heavy metals, from inputs (feed, bedding, water), to system-integrated factors (dust) and outputs (manure). Due to the risk of antibiotic-resistant co-selecting microorganisms, a exploratory approach was conducted to estimate and assess these risks based on higher nutritional concentrations in these environmental samples.

## Material and methods

An online survey and presentations at public events were used to recruit broiler farmers willing to participate in this study. The survey was spread via social media posts and flyers, or through conference contributions and personal contacts with consultants, veterinarians or public institutions. The data collection was conducted in accordance with the guidelines of the Technical University of Munich. Farmers were informed about data protection regulations and the voluntary nature of their participation through the cover letter and the introductory page of the online survey. This information was further acknowledged by signing a data transfer agreement. General management data from the participating farms are presented in Tab. 1. Due to the collection of sensitive information, the publication of the raw data is waived, as agreed upon contractually with the participating farmers. Therefore, raw data are only available from the authors after a reasonable request and signing a data consent form. Additionally, no ethical approval for the use of animals was required, as an examination of living animals or even contact between researchers and animals were excluded.

**Table 1:** General management data of the participating farms (*n* = 14), with the range of numeric data (min and max) in square brackets.

Form of operation	
Conventional, n	10
Organic, n	4
Breeding line	
Fast-growing, n	9
Slow-growing, n	5
Used bedding materials	
Chopped straw, n	4
Straw pellets, n	4
Spelt husks, n	2
Peat, n	1
Spelt pellets, n	1
Wood pellets, n	1
Wood shavings, n	1
Fattening data	
Average number of animals, n	20,079 [500 - 44,000]
Average stocking density, kg/m^3^	29.85 [18 - 39]
Average duration of fattening period, d	52 [37-100]
Average slaughter weight, kg	2.75 [2.5 – 3.0]

### Sampling

Data collection was conducted in Germany between January 2024 and February 2025, where 14 broiler farmers participated in the study. During the last week of an, for the farmer, average fattening period, the participating farmers were asked to collect pooled samples from the fresh finisher feed (∼ 1 kg), the fresh bedding material (∼ 1.5 kg), water from in-barn water lines (∼ 40 mL), manure (250-500 g) and dust (1-5 g) from the broiler house and send to the laboratory. For sample collection, all farmers got an instruction for standardized sampling, and empty, pre-written sampling bags (for feed, bedding material, manure) or sampling tubes (for water and dust). For the purpose of this study, bedding materials are defined as fresh, unused materials, which are spread in the broiler houses prior to the arrival of the day-old chickens, whereas manure is defined as the mixed product of the bedding and the broilers’ excreta, which has accumulated during the fattening period until the sampling day in the bedding (Gerber et al., 2020).

### Analysis

After the samples arrived in the laboratory, water, manure, and dust were aliquoted to conduct either chemical or microbiological analysis. For chemical analyses, feed, bedding and manure samples, original DM was determined (VDLUFA, 2023, 3.1) overnight at 103°C until no more weight loss was detectable (5 g initial weight). Furthermore, for chemical analysis, 200 g of each manure sample was dried overnight at 103°C due to its high moisture content and the risk of spoilage. The remaining manure samples, as well as water and dust samples, were frozen at −18°C until further chemical analyses. Feed, bedding and dried manure were then ground to 1 mm. Every parameter for both the chemical and microbiological tests was carried out in duplicate.

***Chemical analysis***. The water pH (GS 842, Schott, Germany), turbidity (TIR210, VWR, Belgium) and electric conductivity (G1410, Greisinger, Germany) were detected, as well as the pH of feed, bedding and manure samples according to Cassity-Duffey et al. (2015), measuring a 1:10 g/g sample-to-water mixture. For this purpose, the water and manure samples were thawed either at room temperature (for water) or in a refrigerator (for manure).

Nutritional parameters and the fiber components were analyzed according to VDLUFA (2023) only in the feed, bedding and manure samples due to too small amounts and the low expected quantities of water and dust samples. For the chemical analysis, the samples were ground to 1 mm with a feed mill. Then, crude ash (**CA**, VDLUFA 8.1; using a muffle furnace, LT40/11/B180, Nabertherm, Germany), CP (VDLUFA 4.1.1) based on the Kjeldahl method (using Gerhardt Turbotherm and Vapodest 500C system; Gerhardt, Germany), crude fat (**CL**, VDLUFA 5.1.1; using Soxtherm, Gerhardt, Germany) and the fiber components neutral detergent fiber (**aNDFom**, VDLUFA 6.5.1), acid detergent fiber (**ADFom**, VDLUFA 6.5.2) and acid detergent lignin (**ADLom**, VDLUFA 6.5.3) were detected. Furthermore, nitrogen fractions, used as a basis for the estimation of CP, were further specified according to Hesta et al. (2003) or Schwarm et al. (2009). The total amount of nitrogen (**N_tot_**) could therefore be differentiated into undigestible nitrogen (**NDF_SDS_N**) on the one hand, which was detected by first producing the aNDF fraction of the samples (procedure of boiling in neutral detergent without ashing). The resulting indigestible portion was then oven-dried at 103°C until no further weight loss occurred, and the nitrogen content was determined based on the Kjeldahl method. Due to the drying process, a DM of 100% was assumed. NDF_SDS_N was related to the origin sample, and the difference of N_tot_ to NDF_SDS_N resulted in the metabolic nitrogen (**N_met_**), as the second fraction of N_tot_. For statistical analysis, the absolute amounts of NDF_SDS_N and N_met_ were considered, and the proportion of NDF_SDS_N or N_met_ to N_tot_ was calculated.

Additionally, an elemental analysis was conducted in all 5 of the sample types (feed, bedding, water, manure and dust). However, 2 of the 14 dust samples were already used up during the microbiological examination, and the elemental analysis was conducted in the 12 remaining dust samples. For these parameters, both mentioned dust samples were excluded from the statistical analysis. The samples (0.18-0.20 g) were wet-digested with 3 mL of nitric acid (suprapur) in a microwave (MARS 6, CEM Corporation, USA;140°C for 3 min and 200°C for 20 min). Then, the samples were rinsed into falcon tubes, filled up to 50 mL with double-distilled water and again a dilution was made (3 mL solution with 12 mL double-distilled water and 0.7 mL of nitric acid, suprapur). Elements were detected using an ICP-AES (Optima 5300 DV, PerkinElmer, USA, original solution) or an ICP-MS (NexION 300XX, PerkinElmer, USA, diluted sample solution). The resulting values were referred to a conducted blank value and the parallel determined DM. For the dust samples, a DM of 100% and for water of 0% was assumed since the remaining amount did not allow determination of DM. The method was verified by processing digesting-tubes containing 3 mL of nitric acid without sample materials in the microwave blank values. Supplemental Tab. 1 lists all tested elements with their individual limit of quantification (**LOQ**), applicable to the respective sampling solution. If the concentration of a sample was below the LOQ, the LOQ/2 was used for further calculations and statistical analysis.

***Microbiological analysis***. The fresh manure, dust, and water samples were qualitatively tested for the occurrence of *Staphylococci, Staphylococcus aureus* (***S. aureus***), methicillin-resistant *S. aureus* (**MRSA**) and species with resistance to extended-spectrum beta-lactams according to Liegsalz et al. (2026), immediately after arrival in the lab and aliquotation.

Before processing, the manure and dust were diluted in a sterile physiologic saline solution and homogenized. The water samples were used originally. For the occurrence of *Staphylococci* and *S. aureus*, a Baird-Parker selective agar (100063UA, VWR Chemicals, Belgium) was used. MRSA were identified using CHROMagar pre-poured plates (201402, MAST Diagnostica, Reinfeld, Germany) after pre-enrichment in Mueller-Hinton broth (CM0405B, Oxoid Ltd., Thermo Fisher Scientific, USA; supplemented with 6.5% NaCl) and tryptic soy broth (CM0129B, Oxoid Ltd., Thermo Fisher Scientific, USA; including aztreonam (50 mg/L; PHR1785-500MG, Merck KGaA, Germany) and cefoxitin (3.5 mg/L; BIM0108, Apollo Scientific, UK)) for 24 h at 37°C for each broth. The species with resistance to extended-spectrum beta-lactams were isolated using CHROMagar pre-poured plates (201407, MAST Diagnostica, Reinfeld, Germany). After subculturing the individual colonies were isolated on Columbia agar with 5% sheep blood (201190, MAST Diagnostica, Reinfeld, Germany), and identified using mass spectrometry (MALDO-TOF MS, Bruker Daltonics, Germany) with the provided MBT Compass Reference Library. The antibiotic susceptibility tests were conducted according to CLSI guidelines (M07-A10) by both microdilution (Savin et al., 2020) using Micronaut-S MDR MRGN-Screening system (MERLIN, Gesellschaft für mikrobiologische Diagnostika GmbH, Germany) and evaluated according to the European Committee on Antimicrobial Susceptibility Testing (EUCAST) (2025).

### Statistical analysis

After processing the results in Microsoft Excel (2108), statistical analysis was conducted with RStudio (4.5.0) (R Core Team, 2025). Multiple parameters failed the test for normality according to the conducted Shapiro-Wilk test. Therefore, an Aligned Rank Transform (**ART**) modelling (R-package: ARTool) was used, with the sampling types (feed, bedding, water, manure, dust) as fixed factors and the individual farm number as block structure, to determine the differences between the sample types without distributions based on management measures of the individual farmers. This ART modelling was conducted individually for the nutritional parameters and fiber components (in feed, bedding and manure) and for the elemental analysis (for all 5 sample types). Group differences were determined using the Tukey-Kramer test (α < 0.05).

Furthermore, a Firth’s penalized logistic regression (R-package: logistf; Heinze and Schemper (2002) and Puhr et al. (2017)) was conducted as risk analysis. The concentrations of the chemical parameters were, therefore, evaluated for the occurrence or inhibition of the tested microbiological parameters. However, due to the use of metric concentrations in the chemical analyses and the sometimes low occurrence of individual microbiological parameters, the risk analysis could only be conducted for a selection of microbiological parameters: the occurrence of species with resistance to extended-spectrum beta-lactamsin water or dust (excluding manure), in general, biofilm-forming microorganisms in water samples (including *S. aureus*), or the presence of Enterobacteriaceae, *Escherichia coli* (***E. coli***), *Stenotrophomonas maltophilia* (***S. maltophilia***), or multi-resistant microorganisms overall (in water, manure, or dust). Additionally, the Firth’s penalized logistic regression was only conducted for the parameter-relevant predictors (e.g. water predictors for water parameters). The odds ratios (**OR**) could then be calculated based on the estimated effect (β): OR = e^β^. For a higher model validity, correlations of the predictors were evaluated using Pearson correlation test; interactions could not be considered due to the high number of predictors and the relatively small number of samples. P-values below 0.05 were considered significant, and the mean and SE are shown if not mentioned otherwise.

## Results

### Chemical analysis

**Fig. 1:**
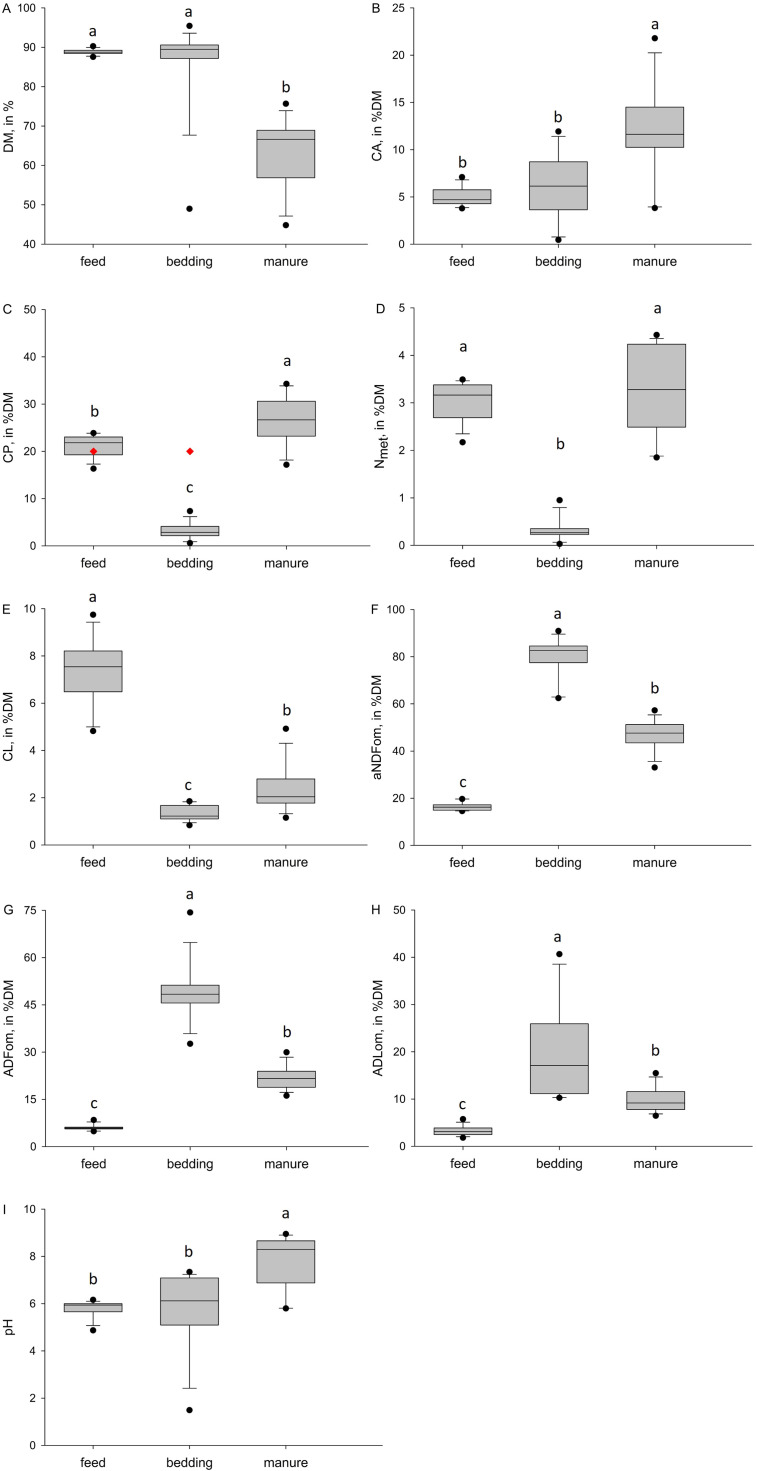
Comparison of the nutritional components, and pH values of different environmental samples (feed, bedding, manure) of broiler farms (*n* = 14). The borders of the boxes indicate the first and third quartiles, with the median as the middle line. Black data points indicate outliers, red diamonds indicate a defined limit based on literature research (feed and bedding according to NRC (1994), water according to Kamphues et al. (2007), manure according to the German Fertilizer Ordinance (*Düngemittelverordnung,* 2017)). ADFom, Acid Detergent Fiber; ADLom, Acid Detergent Lignin; aNDFom, Neutral Detergent Fiber; CA, Crude Ash; CL, Crude Fat; CP, Crude Protein; N_met_, Metabolic Nitrogen.

The water samples showed a mean pH, turbidity and electric conductivity of 7.19 (± 0.092), 1.92 (± 0.741) NTU and 405 (± 61.1) µS, respectively. When the different pH, nutrients and fiber components (aNDFom, ADFom, ADLom) were compared between the sampling types (feed, bedding, manure), differences occurred in each of the parameters (Fig. 1): moisture, CA and CP accumulated from the feed and bedding in the manure, whereas the concentrations of CL and the fiber components of manure ranged between the feed and bedding.

**Fig. 2:**
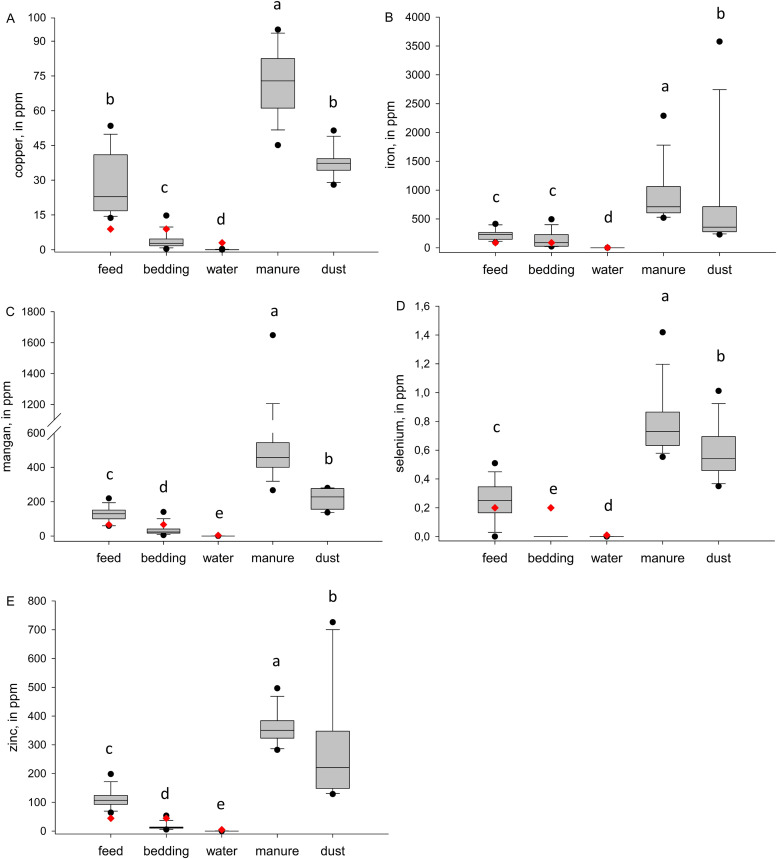
Comparison of the trace element concentration of different environmental samples (feed, bedding, water, manure, dust) of broiler farms (*n* = 14 for feed, bedding, water and manure, *n* = 12 for dust). The borders of the boxes indicate the first and third quartiles, with the median as the middle line. Black data points indicate outliers, red diamonds indicate a defined limit based on literature research (feed and bedding according to NRC (1994), water according to Kamphues et al. (2007), manure according to the German Fertilizer Ordinance (*Düngemittelverordnung*, 2017)).

**Fig. 3:**
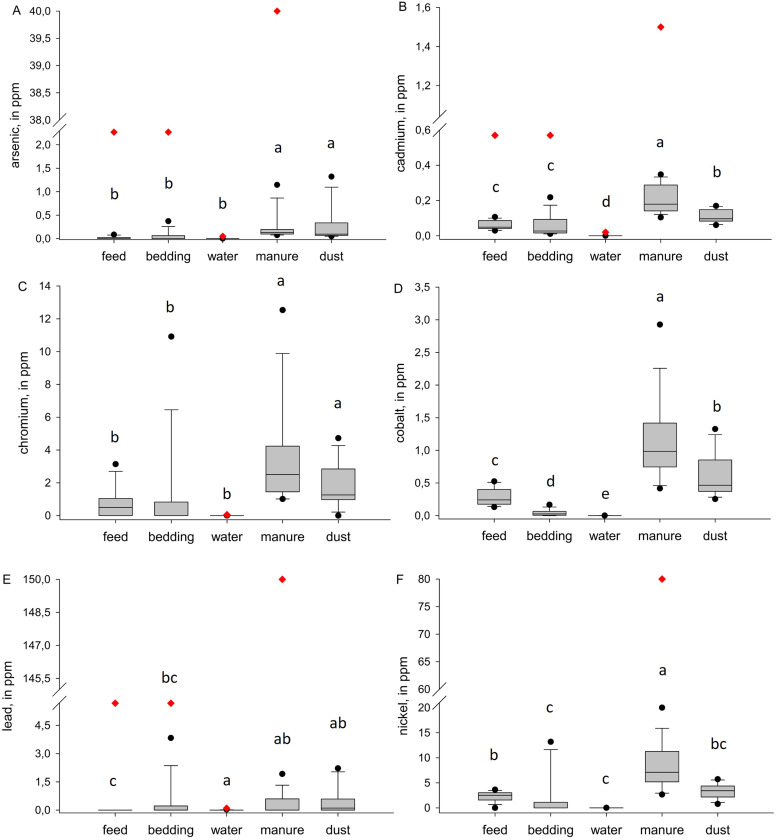
Comparison of the heavy metal concentrations from different environmental samples (feed, bedding, water, manure, dust) of broiler farms (*n* = 14 for feed, bedding, water and manure, *n* = 12 for dust). The borders of the boxes indicate the first and third quartiles, with the median as the middle line. Black data points indicate outliers, red diamonds indicate a defined limit based on literature research (feed and bedding according to NRC (1994), water according to Kamphues et al. (2007), manure according to the German Fertilizer Ordinance (*Düngemittelverordnung,* 2017)).

**Fig. 4:**
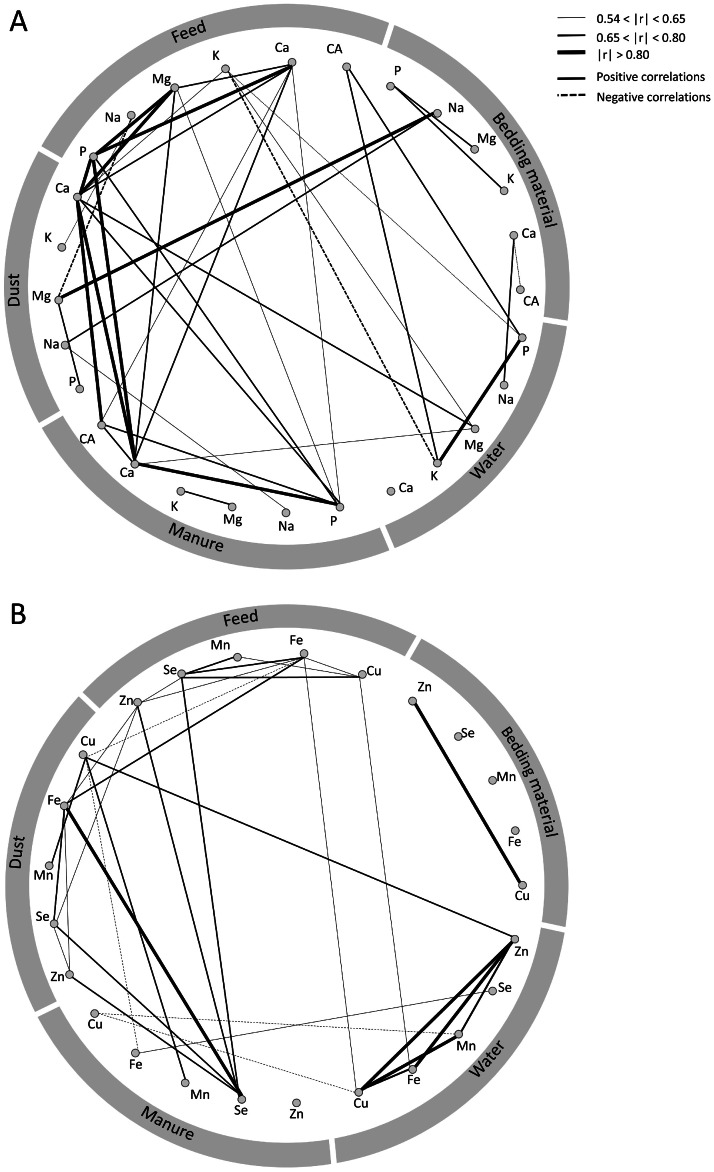
Pearson Correlation Matrix of the major element (A), and trace element (B) concentration. CA, Crude Ash.

The elemental analysis differed significantly between the 5 sample types in all tested elements (supplemental Tab. 1). Sn was only detectable in 2 water and 1 dust sample; in all other samples, the concentration was below LOQ. In addition, the lowest concentrations of trace elements (Fig. 2) and heavy metals (Fig. 3) occurred in the water samples, followed by the feed and bedding. These parameters also accumulated from feed, bedding, and water to manure and dust. Furthermore, the individual major and trace elements correlated within and between the sample types (Fig. 4).

### Microbiological analysis

*Staphylococci* and *S. aureus* were detectable in all manure and dust samples, whereas in the water samples, none (*n* = 3), only *Staphylococci* (*n* = 5) or *S. aureus* (*n* = 1) or both (*n* = 5) occurred. However, MRSA were absent in all of the environmental samples.

**Fig. 5:**
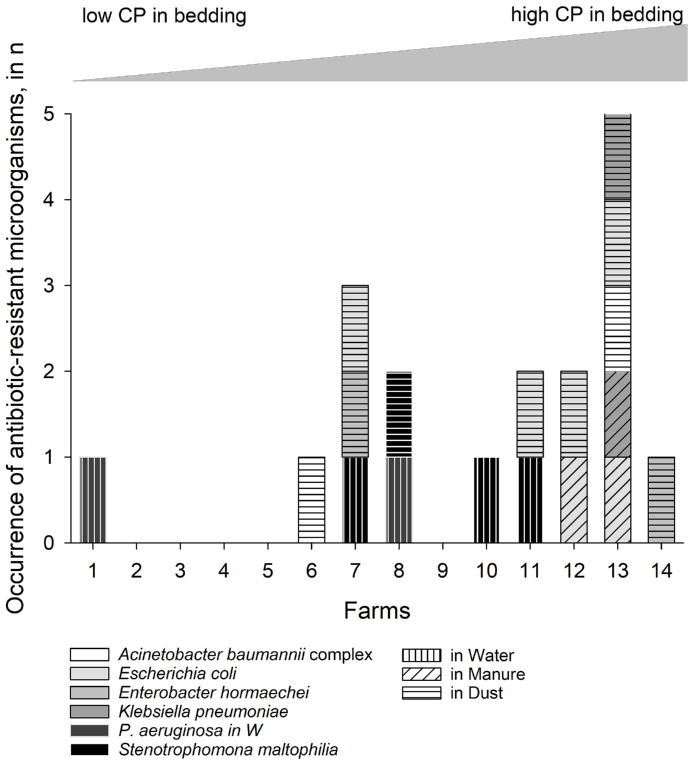
Opportunistic, antibiotic-resistant pathogens occurred in the environmental samples (water, manure, dust) from the participating farms (*n* = 14), sorted based on the concentrations of crude protein (CP) in the corresponding bedding material samples. Microorganisms were detected using CHROMagar for ESBL.

In 9 out of the 14 participating farms, microorganisms occurred on pre-poured agar plates, identifying ESKAPE bacteria and *Stenotrophomonas maltophilia* (Fig. 5). Besides unspecific microorganisms with intrinsic resistances to 3./4. generation of cephalosporins, *Acinetobacter baumannii* complexes (**ABC**, *n* = 2 dust samples), *Enterobacteriaceae*, like *Enterobacter hormaechei* (*n* = 2 dust samples), *E. coli* (*n* = 2 manure and 4 dust samples) or *Klebsiella pneumoniae* (*n* = 1 manure and dust sample each), as well as *Pseudomonas aeruginosa* (*n* = 2 water samples), and *S. maltophilia* (*n* = 3 water and 1 dust sample) occurred in the various environmental samples.

*Enterobacteriaceae* (*n* = 10) showed antibiotic resistance at least to cephalosporine (cefotaxim, ceftazidim) and penicillin (piperacillin). Additionally, some of them (*n* = 4) were also resistant to fluoroquinolones (ciprofloxacin), and therefore, can be classified as resistant to 3 different antibiotic agents (**3MDRO**). One of the ABC was a multidrug-resistant pathogen according to Magiorakos et al. (2012), as it showed resistance to carbapenems (imipenem, meropenem), fluoroquinolones (ciprofloxacin) and the combination of trimethoprim and sulfamethoxazole.

### Risk evaluation

The pH of water, bedding and manure, nutritional parameters, fiber components, and different elements were influential for the risk of the occurrence of various microbiological parameters (Tab. 2). However, intrinsic correlations occurred within and between the sample types, which have to be considered in the context of risk evaluation. CP in bedding has been associated with a higher risk of the occurrence of, e.g., species with resistance to extended-spectrum beta-lactams in dust samples, and multidrug-resistant microorganisms or *Enterobacteriaceae* in all environmental samples (*p* < 0.03). When considering the different nitrogen fractions of the beddings, the absolute amounts of N_met_ also showed a tendency towards a higher risk associated with these parameters (*p* < 0.1). Furthermore, the relative amount of N_met_ (in % of N_tot_) in manure indicated a higher risk for the occurrence of *Enterobacteriaceae* and multidrug-resistant microorganisms (*p* < 0.05). Fiber components (ADFom in bedding (*p* < 0.1) or aNDFom and ADFom in manure (*p* < 0.05)) lowered these risks (Tab. 2). However, it has to be noted that CP in bedding correlated positively with N_met_ (*p*<0.0001, *r* = 0.916), and negatively with ADFom in bedding (*p* < 0.0009, *r* = −0.789) and aNDFom in manure (*p* < 0.05, *r*= −0.534). The aNDFom and ADFom in manure also correlated positively (*p* <*0*.0001, *r* = 0.898) with each other and negatively with the relative amount of N_met_ (*p* < 0.002, *r* =−0.900 and *r*= −0.819 for aNDFom and ADFom, respectively). Additionally, for the occurrence of *S. maltophilia*, the concentration of CL in the bedding seemed to increase (*p* < 0.04), whereas aNDFom in bedding materials also potentially lowered the risk (*p* < 0.1).

**Table 2:** Risk effects of chemical parameters of various environmental samples (feed, bedding, water, manure, dust) on the occurrence of different opportunistic pathogens and antibiotic-resistant patterns.

Predictors	Effects	lower CI	upper CI	Odd ratios	p-Values
Species with resistance to extended-spectrum beta-lactams in dust samples					
CP in bedding	1.06	0.11	3.20	2.88	0.0226
N_met_ in bedding	6.36	−0.35	32.64	577	0.0709
ADFom in bedding	−0.25	−0.92	0.00	0.78	0.0459
Multidrug-resistantmicroorganisms					
pH of water	−4.26	−14.1	0.141	1.15	0.0608
CP in bedding	1.37	0.24	4.12	3.95	0.0081
N_met_ in bedding	4.92	−0.20	20.89	137	0.0620
ADFom in bedding	−0.18	−0.63	0.01	0.84	0.0766
Relative N_met_ in manure 2	0.19	0.01	0.52	1.21	0.0427
aNDFom in manure	−0.21	−0.55	−0.02	0.81	0.0286
ADFom in manue	−0.40	−1.15	−0.01	0.67	0.0437
*Enterobacteriaceae*^1^					
pH of water	−4.26	−14.1	0.141	1.15	0.0608
CP in bedding	1.37	0.24	4.12	3.95	0.0081
N_met_ in bedding	4.92	−0.20	20.89	137	0.0620
ADFom in bedding	−0.18	−0.63	0.01	0.84	0.0766
Relative N_met_ in manure 2	0.19	0.01	0.52	1.21	0.0427
aNDFom in manure	−0.21	−0.55	−0.02	0.81	0.0286
ADFom in manure	−0.40	−1.15	−0.01	0.67	0.0437
*Escherichia coli*^1^					
pH of water	−4.02	−13.9	0.526	1.69	0.0974
*Stenotrophomonas maltophilia*^1^					
pH of bedding	2.13	0.0916	7.99	2960	0.0306
pH of manure	−0.857	−2.15	0.131	1.14	0.0909
CL in bedding	3.84	0.18	8.72	46.75	0.0393
aNDFom in manure	−0.16	−0.42	0.01	0.85	0.0734

ADFom, Acid Detergent Fiber; aNDFom, Neutral Detergent Fiber; CI, Confidence interval; CL, crude fat; CP, crude protein; N_met_, metabolic nitrogen.

1 Isolated from a selective CHROMagarESBL plates and subsequently subjected to antimicrobial susceptibility testing.

2 N_met_, in % of N_tot_ of the sample.

Higher concentrations of the elements in the different environmental sample types, e.g., of thallium in feed, boron in bedding or manure and copper in dust resulted in a significantly higher risk (*p* < 0.05) of the occurrence of various microbiological parameters (Tab. 3). Additionally, multiple other elements in different environmental samples could potentially pose a risk of the microbiological load (*p* < 0.1, *n* = 19), with significant correlations between these predictors. However, thallium in feed, boron in bedding, manure or dust and copper in dust did not correlate with each other (*p* > 0.05).

**Table 3:** Risk effects of element concentration of various environmental samples (feed, bedding, water, manure, dust) on the occurrence of different opportunistic pathogens and antibiotic-resistant patterns.

Predictors	Effects	lower CI	upper CI	Odd ratios	p-Values
Species with resistance to extended-spectrum beta-lactams in dust samples					
Boron in bedding	0.35	0.02	1.12	1.42	0.0292
Sodium in manure	0.00	0.00	0.00	1.00	0.0681
Vanadium in water	−1324.72	−5599.09	161.89	0.00	0.0869
Multidrug-resistant, opportunistic pathogens					
Boron in bedding	1.40	0.11	3.43	30.94	0.0319
Beryllium in bedding	254.65	−10.49	675.40	2.1087×10^293^	0.0606
Chrome in manure	−0.81	−2.77	0.04	1.04	0.0826
Antimony in manure	−100.63	−545.63	2.16	8.71	0.0580
Uranium in manure	−2.16	−6.42	0.12	1.13	0.0665
Copper in dust	0.23	0.00	0.88	2.41	0.0560
Rubidium in dust	−1.17	−3.27	0.05	1.05	0.0627
Uranium in dust	−5.48	−14.87	0.51	1.67	0.0755
*Enterobacteriaceae*^1^					
Boron in bedding	1.40	0.11	3.43	4.06	0.0319
Beryllium in bedding	254.65	−10.49	675.40	3.92×10^110^	0.0606
Chrome in manure	−0.81	−2.77	0.04	0.44	0.0826
Antimony in manure	−100.63	−545.63	2.16	0.00	0.0580
Uranium in manure	−2.16	−6.42	0.12	0.12	0.0665
Copper in dust	0.23	0.00	0.88	1.25	0.0560
Rubidium in dust	−1.17	−3.27	0.05	0.31	0.0627
Uranium in dust	−5.48	−14.87	0.51	0.00	0.0755
*Escherichia coli*^1^					
Phosphorus in manure	−0.0006	−0.0017	0	1.00	0.0741
Cadmium in manure	−15.17	−59.07	1.61	0.00	0.0818
Sodium in manure	0.00	0.00	0.00	1.00	0.0813
Copper in dust	0.24	0.00	0.94	1.27	0.0452
Uranium in dust	−5.40	−15.37	0.92	0.00	0.0986
*Stenotrophomonas maltophilia*^1^					
Aluminum in feed	−0.02	−0.07	0.00	0.98	0.0936
Cadmium in feed	−56.58	−217.95	1.53	0.00	0.0579
Nickel in feed	−0.97	−2.75	0.17	0.38	0.0972
Thallium in feed	188.02	4.60	603.75	4.55×10^8^^1^	0.0434
Potassium in bedding	0.00	0.00	0.00	1.00	0.0942
Boron in manure	0.17	−0.01	0.66	1.19	0.0617
Boron in dust	0.53	0.00	1.42	1.70	0.0500

CI, Confidence interval.

1 Isolated from a selective CHROMagarESBL plates and was subsequently subjected to antimicrobial susceptibility testing.

## Discussion

In this study, the rediscovery of nutritional, fibers, and elemental components of broiler barn inputs (feed, bedding, water) in environmental samples on outputs (dust and manure), and their effects on the occurrence of antibiotic-resistant, opportunistic pathogens were investigated.

### Chemical and microbiological analysis

The concentrations of nutritional factors and fibers were comparable to other studies. The amount of CP in the finisher feed was 21.18±0.60% DM, while the NRC recommends a CP of 18% in finisher feed as fed (90% DM, resulting in 20% CP in DM) (National Research Council, 1994). With a range from 16.32 to 23.87 CP in % DM, some of the animals were slightly oversupplied, which could be reduced without any losses of growth or performance (Such et al., 2021; Lambert et al., 2023). These over-supplementation leads to a comparably high N excretion, which could be determined by de Rauglaudre et al. (2025) to be 45% of the ingested N (CP range in the feed: 18.8-22.6%), performing a meta-analysis. Furthermore, a reduction of CP by 1%-points led to a reduction of N excretion by 0.21 g/d, and, therefore, decreased the environmental volatility (de Rauglaudre et al., 2025). On the other hand, a reduction of CP is only useful and feasible as long as the amino acid (**AA**) profile in the feed is adapted to the broilers’ performance (Lambert et al., 2023). Thus, the concentration of 16.32% DM seems relatively low, although it should be mentioned that these concentrations were fed to slow-growing broilers. Recommendations for these breeding lines are, to the authors' knowledge, currently lacking; however, a more precise assessment of the supply of the broilers can only be made by taking the AA profile into account. Additionally, the examined diets in this study were taken from the last week of fattening, and therefore, represent only the finisher feed and its CP digestibility.

As already mentioned, a reduction of dietary nutritional concentrations to only what is required by the animals is influential for the composition of the manure (de Rauglaudre et al., 2025; Such et al., 2021). However, these compositions also varied in comparison with different studies. Where the CP concentration in the manure samples of this study was on average 26.75±1.39% DM (17.14-34.29% DM), the N_tot_ in Lambert et al. (2023) was 50.4 g/kg DM on day 28 of fattening in the excreta of the broilers fed the control feed (19.5% CP as fed), which could be converted into CP by the factor of 6.25 (VDLUFA, 2023) and would be 31.5 CP in % DM, whereas in Such et al. (2021) the CP concentration in the animals’ excreta was 20.2% DM (32.25 g/kg DM N_tot_) on day 40 of fattening (20.43% CP as fed). Overall, it has to be noted that the comparable studies examined broilers’ excreta, not manure. However, since the concentration of CP in manure showed a trend in correlating with CP in bedding (*p* < 0.1), but was independend of CP in feed (*p* > 0.1), the composition of bedding seems to have a more relevant effect on CP in manure. N_met_ in manure, relative to N_tot_, were, on average 75.12 ± 1.96%, which is comparable to other herbivores in Schwarm et al. (2009), although in the comparative study, also animals’ excreta were tested, not manure. However, based on the emissions of ammonia from animal excrement or manure, it seems useful and necessary to handle and evaluate exhaust air as output from the broilers’ barn, especially when also considering the microbiological load of this air (Heinemann et al., 2020).

Compared to the control feed of Lambert et al. (2023) and Such et al. (2021), the CL concentrations fed by the farmers in this study were relatively low, with 7.43±0.36% DM compared to 8.9% DM (8.0% as fed). However, the National Research Council (1994) recommends values only for linoleic acid and fat-soluble vitamins, but not CL in general. For concentrations of CL in manure, no further investigations have, to the knowledge of the studies’ authors, so far been published. Overall, the bedding materials seemed to play a minor role in the nutrition of broilers, with 3.16±0.60 and 1.32±0.09% DM of CP and CL, although an oral intake of bedding materials is likely low, but not excluded (Malone et al., 1983).

All feed samples of this study exceeded the concentrations of Cu, Fe and Zn, with a minimum of 4.81 (54,1%), 1.65 (1.85%) and 19.9 (44.7%) ppm in DM above the recommendations, respectively, compared to the recommendations published by the National Research Council (1994) (8.9, 88.9 and 44.4 ppm in DM for Cu, Fe and Zn, respectively). These exceedances varied across individual farms and, in some cases, were up to 5 times (for Cu) or 3.5 times (for Fe and Zn) higher than the recommended concentrations. However, for Mn and Se, 2 or 4 out of the 14 feed samples were below the recommendations of 66.7 and 0.2 ppm in DM, respectively, while 1 or 2 of these diets were intended for slow-growing broilers. Also, Nicholson et al. (1999) indicated trace element concentrations in feed were much higher than recommended, although their study only analyzed 3 broiler finisher feeds and compared them with a different, but comparable, recommendation from the Agricultural Research Council (1975). It could be assumed that major and trace elements are fed equally at farm level, either above or below the required concentrations, which could be indicated by several feed and water samples and the conducted Pearson correlation test (Fig. 4). E.g., one sample that showed Se concentrations below the LOQ, also had the lowest concentrations of Cu, Mn and Zn, whereas feed samples showing the highest concentrations of Se had the highest Zn and second-highest Fe concentration. Additionally, the feed sample with the highest Mn also had the highest Cu concentration. A possible explanation for high concentrations of trace elements could be that mineral premixes are supplemented at different levels, affecting all concentrations in parallel and often at levels higher than required to support the growth and performance of the broilers. However, for bedding materials, only a positive correlation was observed between Zn and Cu for trace elements. Missing other correlations could be explained, by the fact that health-supporting additives were mostly supplemented via the feed or water. Bedding materials seems to be more unloaded, natural-based products, so fewer correlations could occur between parameters. Overall, it seems that reducing the trace elements to meet but not exceed the recommendations should be brought more into focus. The upper limits of toxic heavy metals, like As, Cd or Pb (Commission Regulation (EU) No 1275/2013), were not exceeded in the feed or bedding samples.

All water samples were below the recommendations of Kamphues et al. (2007) for the tested physico-chemical parameters, with, e.g., water pH between 5 and 9, electric conductivity below 3,000 µS/cm, and all elements below the recommended concentrations (also shown in Fig. 2, Fig. 3). However, the total microbial load or the load of individual bacteria (e.g., *E. coli*) was not detected in this investigation and should thus be evaluated separately.

A major environmental problem, when heavy metals and trace elements are fed in amounts higher than required, is the accumulation in the outputs and, e.g., soil (Gupta and Charles, 1999; Sheppard and Sanipelli, 2012; Parente et al., 2018). This accumulation in the manure could be detected in this study, where the concentrations of trace elements and heavy metals were significantly higher in manure than in feed, bedding or water. These accumulations were consistent with the results in Sheppard and Sanipelli (2012), calculating the manure-to-feed ratios of different elements. The lowest ratios could be found in Sn with 1.6, followed by Ca and Na (2.0 each), compared to the present results, where Na, P and Ca showed the smallest ratios (1.07, 1.68, 1.97, respectively). Additionally, in both studies, As and Sb showed one of the highest accumulation ratios with 5.5 and 4.0 in Sheppard and Sanipelli (2012) and 14.6 and 5.3 in this study, respectively. It has to be noted that in Sheppard and Sanipelli (2012), it was not specified whether the ratio was estimated on fresh matter (**FM**) or DM. However, when referring to the results (in % FM) of Sheppard and Sanipelli (2012) in relation to DM (assuming 60% DM as in this study and Nicholson et al. (1999)), the absolute concentrations of trace elements in manure of this study were comparable to those from Nicholson et al. (1999) and higher in Sheppard and Sanipelli (2012) . Overall, the amounts of heavy metals in manure were below the upper limits set by of the German Fertilizer Ordinance (Düngemittelverordnung (DüMV, 2017)), appendix 2, Table 1.4). A further reduction would be beneficial due to accumulation in the soil (Gupta and Charles, 1999; Sheppard and Sanipelli, 2012; Parente et al., 2018).

The results of the microbiological analysis, which include the different identified opportunistic pathogens and their resistance profile, were broadly discussed in Liegsalz et al. (2026) and were consistent with other studies, like Heinemann et al. (2020), Younessi et al. (2022) or Tigabie et al. (2025).

### Risk evaluation

When considering the nutritional and fiber components of the inputs, feed played a minor role compared to bedding on manure samples. The effects of CP, N_met_ or CL in bedding or manure were contrary to those of the fiber components (aNDFom in manure and ADFom in bedding and manure). Based on the Pearson correlations, CP, N_met_ and the fiber components influenced each other, which indicates that improved bedding conditions, i.e., higher concentrations of fiber components, could reduce the risk of the occurrence of antibiotic-resistant, opportunistic pathogens, like *Enterobacteriaceae* or multidrug-resistant microorganisms, overall. Considering individual bedding material samples, highest concentrations of CP and lowest concentrations of aNDFom or ADFom could be shown in spelt husk samples (7.33% DM, 62.45% DM, 32.65% DM, respectively), while the lowest concentration of CP and the highest concentrations of aNDFom or ADFom were found in wood pellet samples (0.57% DM, 90.93% DM, 74.33% DM, respectively). This hypothesis of improved bedding material qualities resulting from increased fiber concentration is supported by the investigation of Liegsalz et al. (2025), where positive correlations between fiber components and the WHC of bedding materials could be observed. These higher WHC values could reduce the availability of moisture to microorganisms, and likely the microbial load in the broilers’ environment. However, to support this thesis, other relevant factors, like the water activity or pH of the bedding and, especially, the manure, should also be considered. As described in Payne et al. (2007) or Dunlop et al. (2016), the pH is crucial for the growth kinetics of microorganisms, and should be at least below 7 for bedding materials, although a lower limit should also be considered to establish (Liegsalz et al., 2025). However, the pH of the fresh bedding and the manure in this study had only a minor effect on the occurrence of opportunistic pathogens, which could be explained by the similar characteristics of different bedding materials at the end of the fattening period. While bedding characteristics, such as pH, differ significantly between the original materials (Gontar et al., 2022; Liegsalz et al., 2025), they are comparable at the end of the fattening period. Furthermore, the microbial load generally increases during fattening (Gontar et al., 2022), when the pH in used bedding materials, i.e. manure, is generally higher and DM is generally lower compared to the original material (Gontar et al., 2022). Another aspect which supports the hypothesis of a reduction in CP and an increase in fiber fractions in bedding is the observed risk of relative N_met_ for some microbial parameters. As indicated by Schwarm et al. (2009), N_met_ of manure (or in animals’ excreta) could be further differentiated into endogenic N and bacterial N, where the endogenic N could also serve as a source for microorganisms in the broilers’ environment. These subcategories of endogenic and bacterial N were not applicable to herbivores (Schwarm et al., 2009). Due to the relatively minor proportion of insects or else in the diet of in-house kept broilers this might be also applicable for broilers. The N_met_ fraction was, therefore, not further differentiated in this study, although only further differentiation between endogenic N and bacterial N would have clarified their specific origins and proportions in N_met_, which can originate from broilers’ feed (endogenic N) or from the total microbial load (bacterial N). Therefore, in practical broiler conditions, shifting the N fractions of manure to NDF_SDS_N, might be possible by reducing the CP concentration in the broilers’ feed to their requirement, although N_met_ could also mainly consists of bacterial protein. Overall, and as already mentioned, reducing CP in the inputs would also reduce the environmental impact of the excreted N (de Rauglaudre et al., 2025).

Based on the literature, 10 heavy metals (As, Cd, Co, Cu, Cr, Hg, Mn, Ni, Pb, Zn) could be identified as relevant regarding the occurrence of antibiotic-resistant microorganisms (Seiler and Berendonk, 2012; Nguyen et al., 2019; Mustafa et al., 2021; Shen et al., 2023). However, the risk evaluation indicated only effects in Cd, Cu, Cr, Ni and Pb, where only Cu was associated with an increased risk of the occurrence of resistant *E. coli* and potentially risks of *Enterobacteriaceae* and multidrug-resistant microorganisms. On the contrary, Cd, Cr, and Ni could be associated with a lower risk of microbiological appearances, indicating a sensitivity to these heavy metals (Sterritt and Lester, 1980; Nies, 1999). However, the concentrations of Hg were below the LOQ in nearly all of the samples, which might be due to the wet digestion of the samples in combination of low concentrations and the volutile nature of Hg. Resistances to Mn or Zn were only discussed in the context of Salmonella (Mustafa et al., 2021) or MRSA (Cavaco et al., 2011; Yazdankhah et al., 2014), respectively, which were either not tested or absent in the tested samples. Another minor discussed metal is boron, which was associated with a significantly higher risk of various microbiological parameters. Such associations were also observable in Szadziul et al. (2025), where positive correlations could be shown between boron and antibiotic resistance genes linked to, e.g., aminoglycosides, chloramphenicol, colistin or β-lactam resistances in soil samples. Generally, a confirmation of these results seems useful by specifically considering antibiotic resistance genes or the samples’ resistome. Furthermore, as described in Dróżdż et al. (2020) or Shen et al. (2023), it could be assumed that antibiotic residues could have been found in the environmental samples. Based on that, not only the occurrence of antibiotic-resistant, opportunistic pathogens or the heavy metal concentrations in environmental samples seems to be adequate predictors for the risk assessment, but also the antibiotic residues in environmental samples.

Overall, to assess the risk of co-selection of antibiotic resistance and heavy metals, Seiler and Berendonk (2012) defined the parameter of the minimal heavy metal co-selective concentration (**MCSC**), which was modelled by Arya et al. (2021) for *E. coli* in slurry. Based on the assumption of the fitness cost (carrying the plasmid) by 0.25 and the different death rate between sensitive and resistant cells (1.25), Arya et al. (2021) estimated, e.g., the MCSC for Cu (5.5 mg/L), Zn (1.6 mg/L) or Pb (21.5 mg/L) in slurry, which would be considered very low even taking into account the low DM of slurry (5.22-7.66% according to Hindrichsen et al. (2005) and Regueiro et al. (2016)). However, the defined MCSC appears to be a promising parameter and should be further investigated to reduce the risk of co-selected microorganisms in the environment.

Although this investigation yielded various conclusions regarding the composition of, especially, bedding materials, these results should be verified accordingly. Therefore, a larger number of participating farms would be helpful to confirm these risks, or findings should serve as a basis for future hypothesis development. In particular, experiments to further investigate the correlations of CP, aNDFom and N_met_ in bedding materials and their effects on antibiotic-resistant microorganisms should be conducted. Additionally, the detection of antibiotic-resistance genes or resistomes, in general, could also confirm the indicated risks posed by different heavy metals.

In conclusion, this study investigated and evaluated nutritional and fiber environmental components (heavy metals or opportunistic pathogens) in inputs (feed, bedding, water) of broiler fattening systems and their accumulation in outputs (manure and dust). In comparison between farms, it seemed that a reduction of CP and trace elements could be feasible without falling below the recommended, required concentrations. Based on the conducted risk evaluation, concentrations of CP in bedding could increase the risk of antibiotic-resistant, opportunistic pathogens, while fiber components in bedding or manure lowered this risk. Especially the relative amount N_met_ to N_tot_ in manure could play a crucial role to predict the environmental colonisation of microorganisms and could, therefore, potentially function as an indicator. To verify this hypothesis, further investigationa are needed.

## Funding sources

This work (Model- and Demonstration Project for animal welfare) is financially supported by the Federal Ministry of Agriculture, Food and Regional Identity (BMLEH) based on a decision of the Parliament of the Federal Republic of Germany, granted by the Federal Office for Agriculture and Food (BLE); grant number 2820MDT220.

## CRediT authorship contribution statement

**Theresa M Liegsalz:** Conceptualization, Data curation, Formal analysis, Investigation, Methodology, Software, Validation, Visualization, Writing – original draft, Writing – review & editing. **Mykhailo Savin:** Data curation, Formal analysis, Methodology, Validation, Writing – review & editing. **Uwe Blum:** Data curation, Formal analysis, Methodology, Validation, Writing – review & editing. **Céline Heinemann:** Conceptualization, Funding acquisition, Methodology, Supervision, Validation, Writing – review & editing. **Julia Steinhoff-Wagner:** Conceptualization, Data curation, Formal analysis, Funding acquisition, Methodology, Project administration, Resources, Supervision, Validation, Visualization, Writing – review & editing.

## Disclosures

We have no conflicts of interest to disclose, and the results have not been published in any other way, nor is the manuscript currently under consideration for publication. The current manuscript should be used for a doctoral project at the TUM School of Life Sciences. Additionally, the manuscript was written through contributions of all authors. All authors have given approval to the final version of the manuscript.

## Appendix Supplementary materials

Image, application 1

Image, application 2

## Footnotes

Supplementary material associated with this article can be found, in the online version, at doi:10.1016/j.psj.2026.106819.
