# Supplementary material for: Integrated risk evaluation of in- and outputs, including heavy metals, in broiler farm environments for the appearance of antimicrobial resistance
Source: Poult Sci. 2026 Mar 18;105(6):106819. doi: 10.1016/j.psj.2026.106819 (PMC13054082; doi:10.1016/j.psj.2026.106819)

## Slide 1
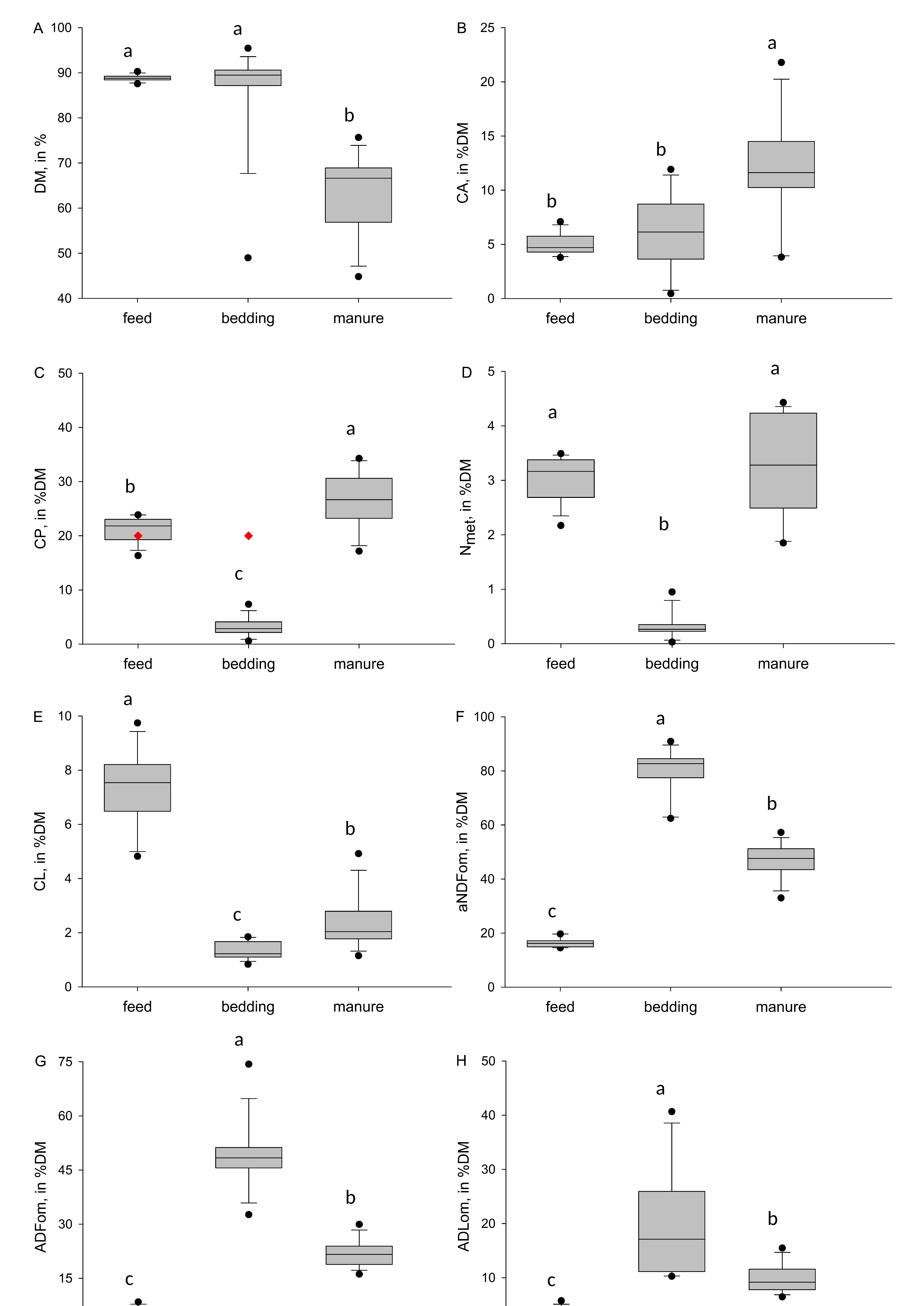

a
a
a
b
b
b
a
a
a
b
b
c
a
a
b
b
c
c
a
a
b
b
c
c
a
b
b

## Slide 2
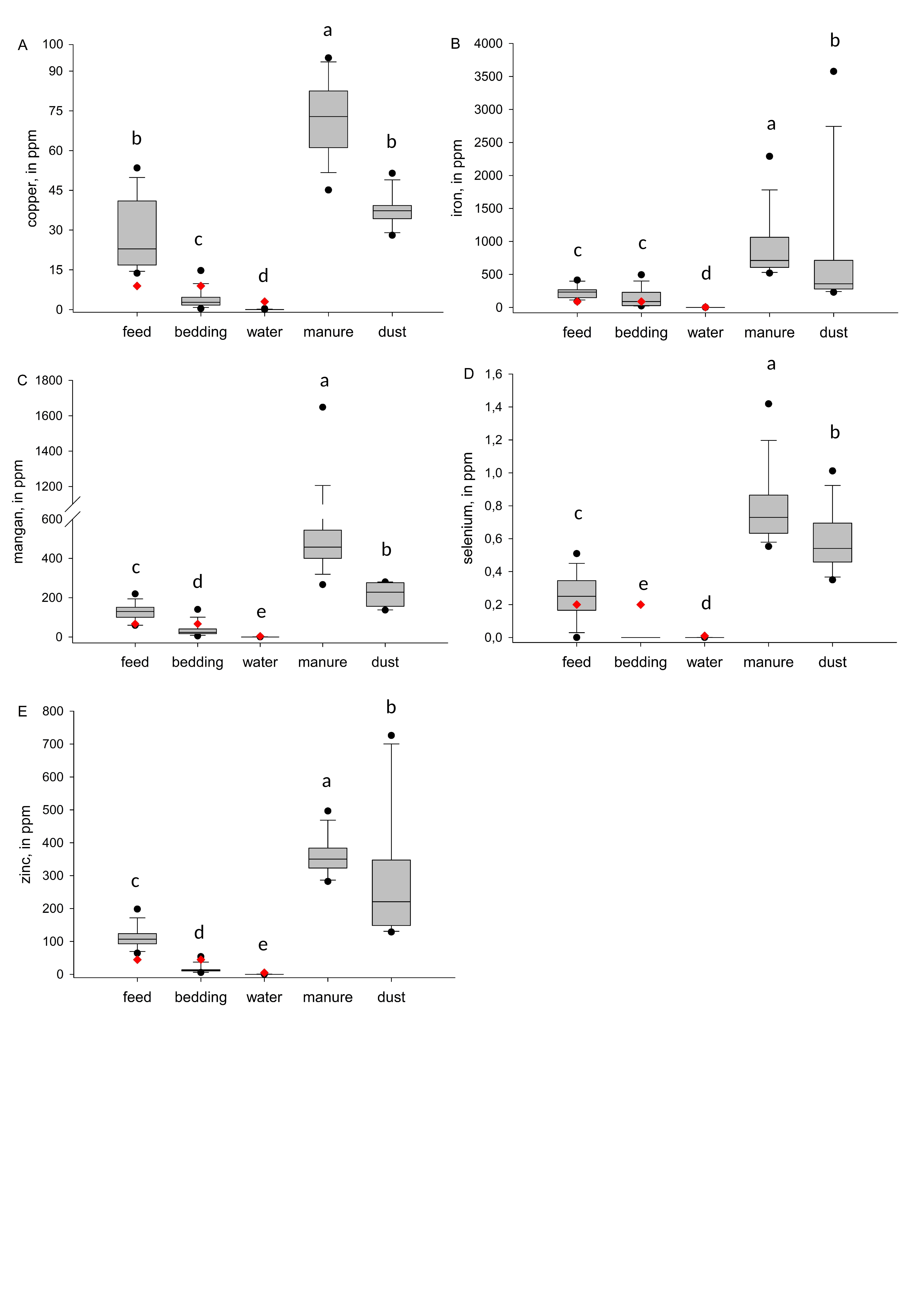

a
b
a
b
b
c
c
c
d
d
a
a
b
c
b
c
d
e
d
e
b
a
c
d
e

## Slide 3
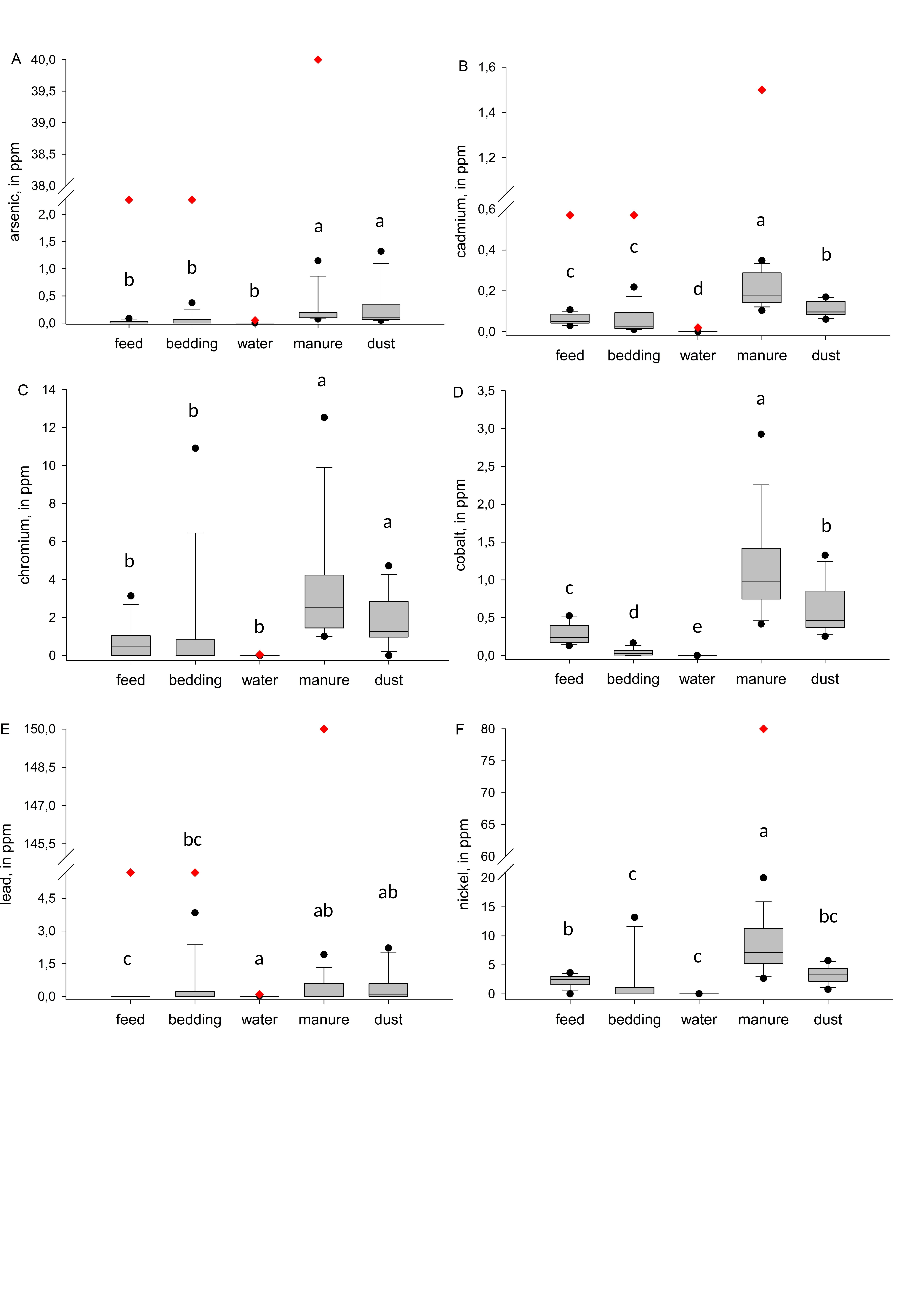

a
a
a
c
b
b
c
b
d
b
a
a
b
a
b
b
c
d
e
b
a
bc
c
ab
ab
bc
b
c
a
c

## Slide 4
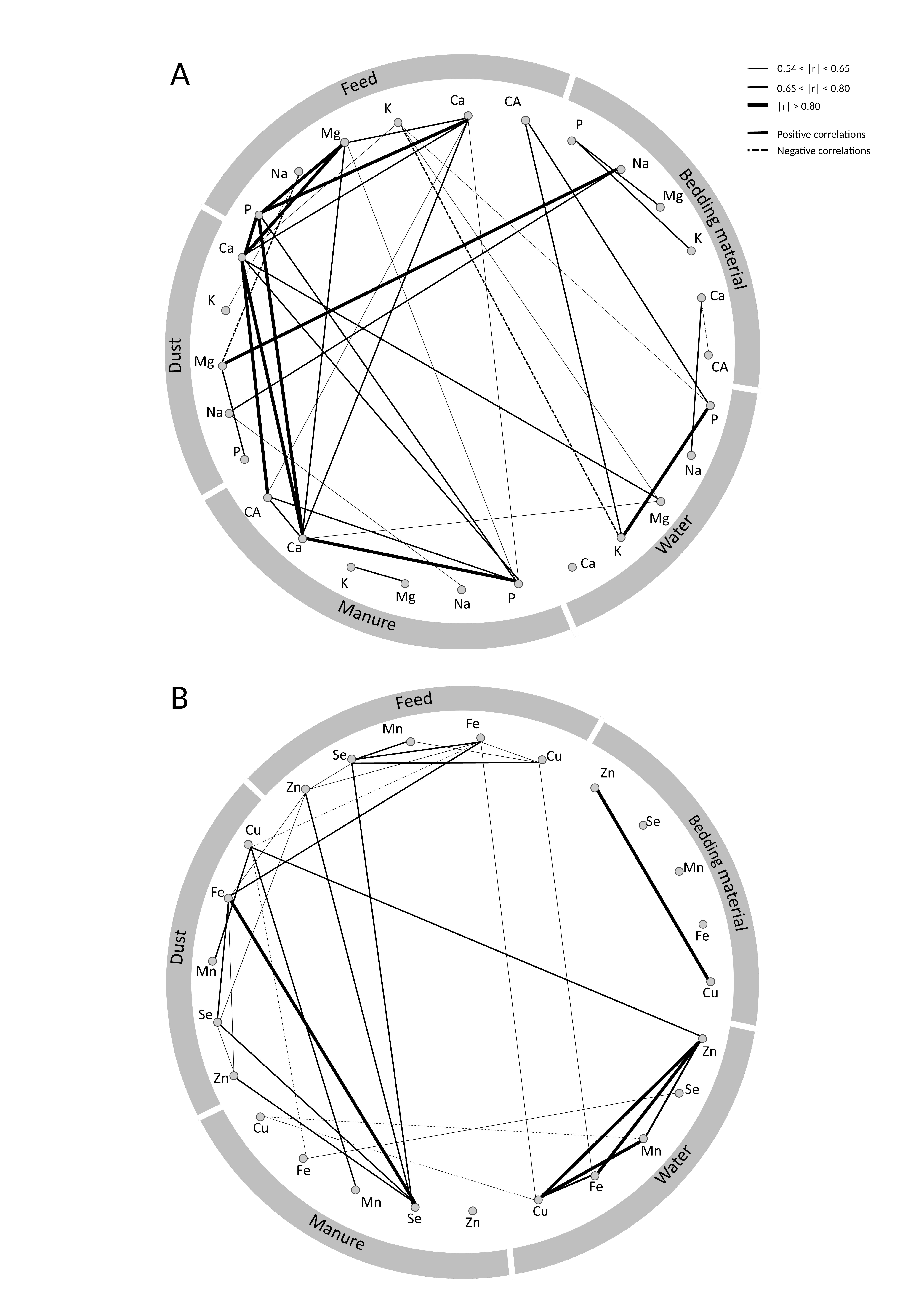

A
0.54 < |r| < 0.65
0.65 < |r| < 0.80
|r| > 0.80
Positive correlations
Negative correlations
B

## Slide 5
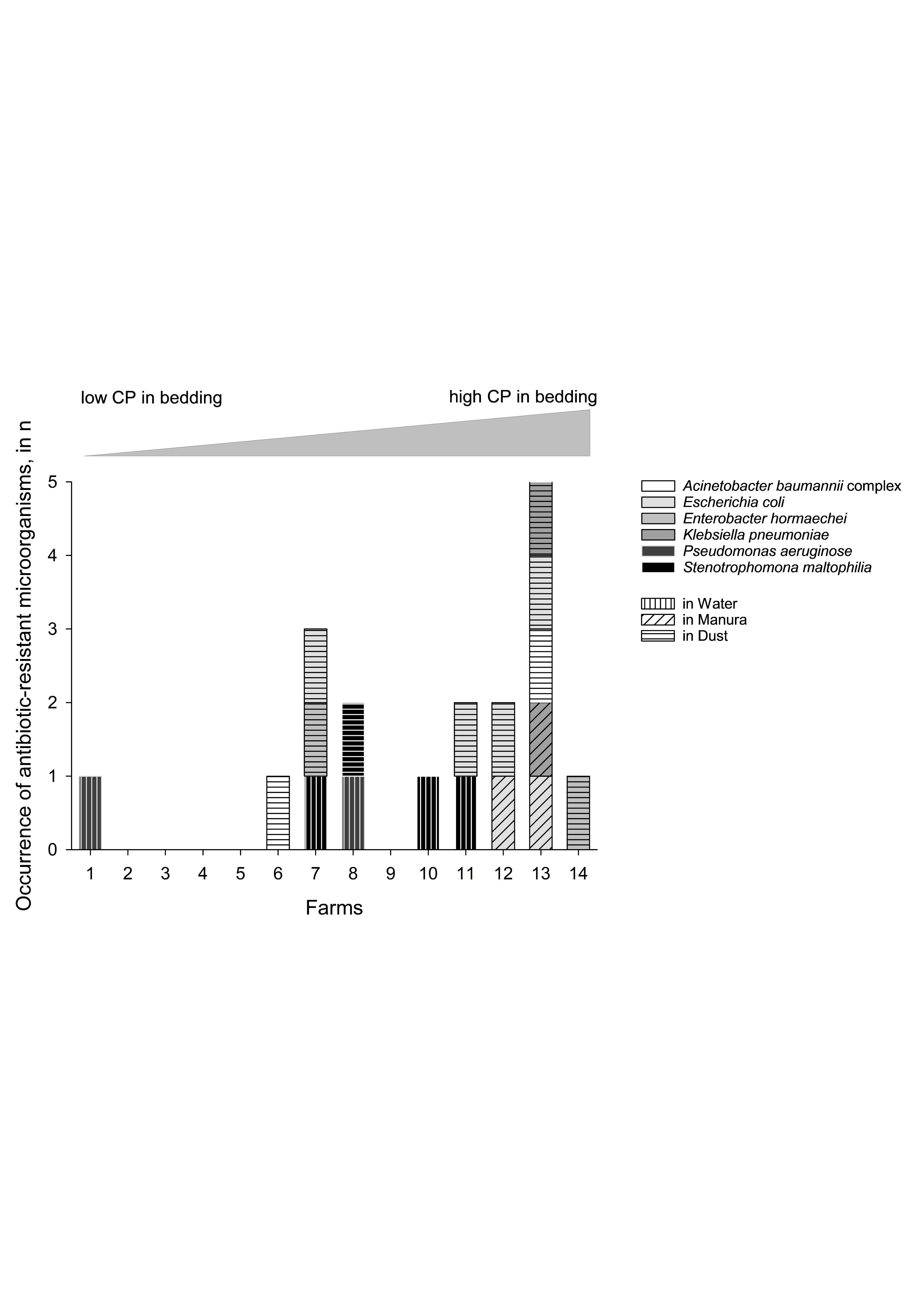

Supplement: Supplementary file 1 [file mmc1.pptx]
