# Supplementary material for: Integrated risk evaluation of in- and outputs, including heavy metals, in broiler farm environments for the appearance of antimicrobial resistance
Source: Poult Sci. 2026 Mar 18;105(6):106819. doi: 10.1016/j.psj.2026.106819 (PMC13054082; doi:10.1016/j.psj.2026.106819)
Supplement: Supplementary file 2 [file mmc2.docx]

Supplemental Table 1: Results and differences of the elemental analysis based on the ART modelling between the different sample types, in ppm

|  | LOQ* | Feed (n = 14) | | |  | Bedding (n = 14) | | |  | Water (n = 14) | | |  | Manure (n = 14) | | |  | Dust (n = 12) | | | p-values |
| --- | --- | --- | --- | --- | --- | --- | --- | --- | --- | --- | --- | --- | --- | --- | --- | --- | --- | --- | --- | --- | --- |
|  |  | mean |  | SE |  | mean |  | SE |  | mean |  | SE |  | mean |  | SE |  | mean |  | SE |  |
| Analyzed by ICP-AES |  |  |  |  |  |  |  |  |  |  |  |  |  |  |  |  |  |  |  |  |  |
| Aluminum (Al) | 3 | 146 | ± | 24.0^c^ |  | 114 | ± | 38.4^c^ |  | 0.0474 | ± | 0.0073^d^ |  | 772 | ± | 158^a^ |  | 483 | ± | 218^b^ | < .0001 |
| Calcium (Ca) | 20 | 8210 | ± | 930^b^ |  | 3574 | ± | 1170^c^ |  | 62.2 | ± | 7.57^d^ |  | 16199 | ± | 2779^a^ |  | 8700 | ± | 2043^b^ | < .0001 |
| Potassium (K) | 3 | 9179 | ± | 253^c^ |  | 9594 | ± | 1968^c^ |  | 2.11 | ± | 0.531^d^ |  | 26259 | ± | 982^a^ |  | 17349 | ± | 1385^b^ | < .0001 |
| Magnesium (Mg) | 1 | 2064 | ± | 91.4^c^ |  | 578 | ± | 54.0^d^ |  | 13.5 | ± | 2.97^e^ |  | 6658 | ± | 247^a^ |  | 4569 | ± | 275^b^ | < .0001 |
| Manganese (Mn) | 0.6 | 126 | ± | 11.4^c^ |  | 33.6 | ± | 9.05^d^ |  | 0.8350 | ± | 0.0556^e^ |  | 551 | ± | 90.1^a^ |  | 219 | ± | 16.0^b^ | < .0001 |
| Phosphorus (P) | 20 | 5589 | ± | 275^b^ |  | 884 | ± | 141^c^ |  | 0.106 | ± | 0.4690^d^ |  | 9367 | ± | 911^a^ |  | 5237 | ± | 325^b^ | < .0001 |
| Sulfur (S) | 20 | 3015 | ± | 156^c^ |  | 3724 | ± | 2900^b^ |  | 12.4 | ± | 1.84^d^ |  | 6608 | ± | 386^a^ |  | 7173 | ± | 546^a^ | < .0001 |
| Zinc (Zn) | 1 | 111 | ± | 8.77^c^ |  | 14.9 | ± | 3.12^d^ |  | 0.0955 | ± | 0.0661^e^ |  | 361 | ± | 159^a^ |  | 289 | ± | 56.9^b^ | < .0001 |
| Analyzed by ICP-MS |  |  |  |  |  |  |  |  |  |  |  |  |  |  |  |  |  |  |  |  |  |
| Arsenic (As) | 0.05 | 0.0162 | ± | 0.0073^b^ |  | 0.0483 | ± | 0.0273^b^ |  | 0.0007 | ± | 0.0001^b^ |  | 0.237 | ± | 0.0788^a^ |  | 0.264 | ± | 0.106^a^ | < .0001 |
| Boron (B) | 2 | 8.89 | ± | 0.502^c^ |  | 0.502 | ± | 0.231^d^ |  | 0.0163 | ± | 0.0033^d^ |  | 30.1 | ± | 2.02^a^ |  | 11.8 | ± | 0.711^b^ | < .0001 |
| Barium (Ba) | 3 | 5.25 | ± | 1.43^b^ |  | 15.5 | ± | 3.84^a^ |  | 0.0261 | ± | 0.0050^c^ |  | 19.0 | ± | 1.60^a^ |  | 10.5 | ± | 1.60^a^ | < .0001 |
| Beryllium (Be) | 0.0007 | 0.0102 | ± | 0.0026^b^ |  | 0.0030 | ± | 0.0011^c^ |  | 0.0000 | ± | 0.0000^c^ |  | 0.0533 | ± | 0.0143^a^ |  | 0.0206 | ± | 0.0066^b^ | < .0001 |
| Bism (Bi) | 0.002 | 0.0006 | ± | 0.0002^b^ |  | 0.0040 | ± | 0.0019^b^ |  | 0.0000 | ± | 0.0000^b^ |  | 0.0077 | ± | 0.0014^a^ |  | 0.0197 | ± | 0.0060^a^ | < .0001 |
| Cadmium (Cd) | 0.003 | 0.060 | ± | 0.0067^c^ |  | 0.5760 | ± | 0.1610^c^ |  | 0.0000 | ± | 0.0000^d^ |  | 0.2072 | ± | 0.0215^a^ |  | 0.108 | ± | 0.1050^b^ | < .0001 |
| Cobalt (Co) | 0.008 | 0.285 | ± | 0.0361^c^ |  | 0.0435 | ± | 0.1280^d^ |  | 0.0002 | ± | 0.0001^e^ |  | 1.13 | ± | 0.168^a^ |  | 0.613 | ± | 0.9400^b^ | < .0001 |
| Chromium (Cr) | 0.5 | 0.742 | ± | 0.254^b^ |  | 1.07 | ± | 0.773^b^ |  | 0.0003 | ± | 0.0000^b^ |  | 3.58 | ± | 0.847^a^ |  | 1.74 | ± | 0.383^a^ | < .0001 |
| Copper (Cu) | 0.5 | 27.2 | ± | 3.48^b^ |  | 3.69 | ± | 0.930^c^ |  | 0.0307 | ± | 0.0232^d^ |  | 72.5 | ± | 3.73^a^ |  | 37.5 | ± | 1.71^b^ | < .0001 |
| Iron (Fe) | 4 | 233 | ± | 24.2^c^ |  | 141 | ± | 37.5^c^ |  | 0.3450 | ± | 0.0179^d^ |  | 887 | ± | 125^a^ |  | 690 | ± | 268^b^ | < .0001 |
| Mercury (Hg) | 0.004 | < LOQ | | |  | 0.0007 | ± | 0.0004^a^ |  | 0.0000 | ± | 0.0000^b^ |  | 0.0006 | ± | 0.0004^a^ |  | 0.0006 | ± | 0.0006^a^ | < .0001 |
| Molybdenum (Mo) | 0.02 | 1.40 | ± | 0.228^b^ |  | 0.608 | ± | 0.154^c^ |  | 0.0004 | ± | 0.0001^d^ |  | 4.64 | ± | 0.370^a^ |  | 1.86 | ± | 0.232^b^ | < .0001 |
| Sodium (Na) | 3 | 3999 | ± | 857^ab^ |  | 2966 | ± | 2473^c^ |  | 22.6 | ± | 7.66^d^ |  | 4293 | ± | 331^a^ |  | 2835 | ± | 394^b^ | < .0001 |
| Nickel (Ni) | 1 | 2.35 | ± | 0.257^b^ |  | 1.98 | ± | 1.15^c^ |  | 0.0011 | ± | 0.0006^c^ |  | 8.20 | ± | 1.23^a^ |  | 3.40 | ± | 0.412^ab^ | < .0001 |
| Lead (Pb) | 0.4 | < LOQ | | |  | 0.400 | ± | 0.277^a^ |  | 0.0026 | ± | 0.0022^b^ |  | 0.338 | ± | 0.144^ab^ |  | 0.449 | ± | 0.210^ab^ | < .0005 |
| Ruthenium (Ru) | 0.02 | 5.26 | ± | 0.177^d^ |  | 1.59 | ± | 0.232^c^ |  | 0.0008 | ± | 0.0002^e^ |  | 10.9 | ± | 0.415^a^ |  | 7.77 | ± | 0.356^b^ | < .0001 |
| Antimony (Sb) | 0.01 | 0.0036 | ± | 0.0021^bc^ |  | 0.0051 | ± | 0.0051^b^ |  | 0.0000 | ± | 0.0000^c^ |  | 0.0189 | ± | 0.0047^a^ |  | 0.0529 | ± | 0.0323^a^ | < .0001 |
| Selenium (Se) | 0.1 | 0.253 | ± | 0.369^c^ |  | < LOQ | | |  | 0.0002 | ± | 0.0001^d^ |  | 0.770 | ± | 0.0587^a^ |  | 0.578 | ± | 0.0511^b^ | < .0001 |
| Tin (Sn) | 0.09 | < LOQ | | |  | < LOQ | | |  | < LOQ | | |  | < LOQ | | |  | < LOQ | | |  |
| Strontium (Sr) | 0.06 | 13.0 | ± | 2.35^bc^ |  | 8.18 | ± | 1.55^c^ |  | 0.245 | ± | 0.0537^d^ |  | 34.7 | ± | 4.46^a^ |  | 17.0 | ± | 1.90^b^ | < .0001 |
| Titanium (Ti) | 0.1 | 4.23 | ± | 0.915^b^ |  | 2.02 | ± | 0.786^c^ |  | 0.0004 | ± | 0.0001^d^ |  | 12.8 | ± | 2.53^a^ |  | 9.61 | ± | 4.77^ab^ | < .0001 |
| Thallium (Tl) | 0.0001 | 0.015 | ± | 0.0019^b^ |  | 0.0063 | ± | 0.0021^c^ |  | 0.0000 | ± | 0.0000^d^ |  | 0.0380 | ± | 0.0042^a^ |  | 0.0353 | ± | 0.0058^a^ | < .0001 |
| Uranium (U) | 0.001 | 0.127 | ± | 0.005^b^ |  | 0.0065 | ± | 0.0030^c^ |  | 0.0017 | ± | 0.0006^c^ |  | 0.669 | ± | 0.156^a^ |  | 0.340 | ± | 0.0604^ab^ | < .0001 |
| Vanadium (V) | 0.003 | 0.100 | ± | 0.0823^b^ |  | 0.0104 | ± | 0.0062^b^ |  | 0.0006 | ± | 0.0002^ab^ |  | 1.26 | ± | 0.605^a^ |  | 0.668 | ± | 0.411^ab^ | < 0.001 |

*in µg/L; based on the sampled solution, not the specified value (as stated for feed, bedding, water, manure or dust), which has already been related to the DM and for which the mean values of the two duplicates were already calculated.
LOQ, Limit of quantification
